# Effect of desflurane anesthesia on flash visual evoked potential monitoring in patients undergoing spine surgery: study protocol for a randomized controlled trial

**DOI:** 10.1186/s13063-024-08211-9

**Published:** 2024-06-06

**Authors:** Jiajia Ma, Jing Wang, Yun Li, Yuxuan Fu, Yang Li, Hui Qiao, Ruquan Han

**Affiliations:** 1 Department of Anaesthesiology, Beijing Tiantan Hospital, Capital Medical University, No. 119, Southwest 4th Ring Road, Fengtai District, Beijing, PR China; 2 Department of Electrophysiology, Beijing Neurosurgical Institute, Beijing, 100070 People’s Republic of China

**Keywords:** Flash visual evoked potentials, Desflurane, Propofol, Balanced anesthesia, Spine surgery

## Abstract

**Background:**

Flash visual evoked potentials (FVEPs) are a reliable method for protecting visual function during spine surgery in prone position. However, the popularization and application of FVEPs remain limited due to the unclear influence of various anesthetics on FVEPs. Exploring the effects of anesthetic drugs on FVEP and establishing appropriate anesthesia maintenance methods are particularly important for promoting and applying FVEP. According to the conventional concept, inhaled narcotic drugs significantly affect the success of FVEP monitoring, FVEP extraction, and interpretation. Nonetheless, our previous study demonstrated that sevoflurane-propofol balanced anesthesia was a practicable regimen for FVEPs. Desflurane is widely used in general anesthesia for its rapid recovery properties. As the effect of desflurane on FVEP remains unclear, this trial will investigate the effect of different inhaled concentrations of desflurane anesthesia on amplitude of FVEPs during spine surgery, aiming to identify more feasible anesthesia schemes for the clinical application of FVEP.

**Methods/ design:**

A total of 70 patients undergoing elective spinal surgery will be enrolled in this prospective, randomized controlled, open-label, patient-assessor-blinded, superiority trial and randomly assigned to the low inhaled concentration of desflurane group (LD group) maintained with desflurane-propofolremifentanil-balanced anesthesia or high inhaled concentration of desflurane group (HD group) maintained with desflurane-remifentanil anesthesia maintenance group at a ratio of 1:1. All patients will be monitored for intraoperative FVEPs, and the baseline will be measured half an hour after induction under total intravenous anesthesia (TIVA). After that, patients will receive 0.5 minimum alveolar concentration (MAC) of desflurane combined with propofol and remifentanil for anesthesia maintenance in the LD group, while 0.7–1.0 MAC of desflurane and remifentanil will be maintained in the HD group. The primary outcome is the N75-P100 amplitude 1 h after the induction of anesthesia. We intend to use the dual measure evaluation, dual data entry, and statistical analysis by double trained assessors to ensure the reliability and accuracy of the results.

**Discussion:**

This randomized controlled trial aims to explore the superiority effect of low inhaled concentration of desflurane combined with propofolremifentanil-balanced anesthesia versus high inhaled concentration of desflurane combined with remifentanil anesthesia on amplitude of FVEPs. The study is meant to be published in a peer-reviewed journal and might guide the anesthetic regimen for FVEPs. The conclusion is expected to provide high-quality evidence for the effect of desflurane on FVEPs and aim to explore more feasible anesthesia schemes for the clinical application of FVEPs and visual function protection.

**Trial registration:**

This study was registered on clinicaltrials.gov on July 15, 2022. ClinicalTrials.gov Identifier: NCT05465330.

**Supplementary Information:**

The online version contains supplementary material available at 10.1186/s13063-024-08211-9.

## Strengths and limitations of this study

- This prospective, randomized, controlled, open-label, patient-assessor-blinded trial aims to investigate the superiority effect of low inhaled concentration of desflurane combined with propofolremifentanil-balanced anesthesia versus high inhaled concentration of desflurane combined with remifentanil anesthesia on amplitude of FVEPs during spine surgery.
- This study will target a pragmatic endpoint, and the conclusion is expected to provide high-quality evidence for the effect of desflurane on FVEPs, aiming to explore more feasible anesthesia schemes for the clinical application of FVEPs and visual function protection. Notably, we intend to use the dual measure evaluation, dual data entry, and statistical analysis by double trained assessors to ensure the reliability and accuracy of the results.
- This study focuses on the effect of desflurane on FVEPs, and further studies could evaluate other inhalations or new anesthetics.

## Background

Loss of vision during prone surgery is a devastating complication that occurs due to a variety of reasons [1]. Intraoperative prone position can cause mechanical damage or ischemia of visual structures, causing postoperative visual loss or visual field defects [2]. The flash stimulus is transmitted from the retina to the optic nerve, optic chiasm, optic tract, lateral geniculate body, optic radiation, and visual cortex and converted from a light signal to electrical conduction. The evoked potential waveforms and data extracted from the occipital lobe area during surgery are called FVEPs. They reflect the integrity of the visual pathway in real time to guide intraoperative decision-making and prevent visual impairment or deterioration of the same caused by visual pathway damage [3–5]. Previous studies have shown that the amplitude is the main measure for evaluating FVEP, in addition to the latency value. Recently, a study showed that setting the early warning value as the FVEPs’ amplitude decreased by 20% from the baseline can detect new quadrant blindness [6]. FVEPs have become a reliable method for intraoperative protection of visual function in procedures jeopardizing visual pathway integrity or visual function, such as spine surgery in the prone position, neurosurgery of the sellar region, and similar.

Anesthetic drugs dramatically affect the amplitude and latency of FVEP and can even lead to the failure of FVEP extraction and interpretation, which limits the popularization and application of FVEPs. Exploring the effects of anesthetic drugs on FVEP and establishing appropriate anesthesia maintenance methods is particularly important for promoting and applying FVEP to promote perioperative visual function protection. Previously, it was commonly believed that neuromonitoring should be performed under total intravenous anesthesia and that inhalation anesthetics could significantly prolong the latency and reduce the amplitudes of evoked potentials, not confined solely to FVEPs but also extending to somatosensory evoked potential (SSEP) and motor-evoked potential (MEP) [7–10]. In their study, Reinacher et al. found that as the concentration of sevoflurane increased incrementally below 0.5 MAC, 0.75 MAC, and 1 MAC, the amplitude of MEP decreased in a concentration-dependent manner [11]. Similarly, Boisseau obtained consistent results in the intraoperative impact of sevoflurane on SSEP [12]. Vaugha et al. observed that sevoflurane and desflurane exhibited an increasing inhibitory effect on SSEP with increasing concentrations, reaching maximum inhibition at end-tidal concentrations of 3.2 and 4.9%, respectively [13]. Accordingly, TIVA was preferred for maintenance. However, some studies showed that the P100 amplitude was significantly reduced at plasma concentrations of 3.0 μg/ml propofol compared to 1.5 μg/ml, even during TIVA [14]. Besides, TIVA may not be suitable for lengthy surgeries needing motor-evoked potential monitoring with strict restriction for muscle relaxant supplements, especially during long-term spine surgery or neurosurgery.

Appropriate use of inhalation anesthetics, such as intravenous-inhalation balanced anesthesia, can significantly reduce the dosage of each type of anesthetic and may reduce the adverse effects of high-dose inhalation anesthetics or intravenous narcotics on electrophysiological monitoring [15]. Specifically, propofol acts mainly by activating gamma-aminobutyric acid receptors (GABA). In contrast, inhalation anesthetics act on N-methyl-D-aspartic acid receptors (NMDA) before and after synapses through various pathways, glycine, kainate, serotonin receptors, and potassium channels in addition to GABA, nicotinic choline, and calcium channels, which have a certain synergy between them. However, in 2017, Uribe et al. proposed that compared with TIVA, intravenous-inhalation balanced anesthesia based on desflurane significantly reduced the amplitude of FVEPs and prolonged their latency [16]. Conversely, our recent study showed that the effect of sevoflurane 0.5 MAC combined with propofol and remifentanil-balanced anesthesia on P100-N145 amplitude was not inferior to propofol-based TIVA. It suggests that sevoflurane-propofol balanced anesthesia is suitable as an optional anesthesia regimen for FVEPs [17]; however, the effect of inhalation at different concentrations on FVEPs remains controversial [16–18].

Desflurane is widely used in general anesthesia for its rapid recovery properties and unique advantages [19], including a low blood-gas partition coefficient, faster recovery of postoperative swallowing function and airway protective reflex, and high quality of recovery, conducive to the early recovery of respiratory function and orientation, in addition to early neurological evaluation. Furthermore, desflurane can significantly increase and prolong the muscle relaxant effect of nondepolarizing muscle relaxants compared with intravenous anesthesia [20]. For patients who need motor-evoked potential monitoring simultaneously, it can reduce the intraoperative muscle relaxant dosage and the incidence of intraoperative body movement and choking [21, 22], contributing to its wide use in general anesthesia during spine surgery. Until April 2024, 10 RCTs exploring the effects of anesthetics on FVEPs have been published, and only one of them involved desflurane, while high-concentration desflurane was not involved. A review of the Cochrane Library conducted in 2011, attempting to assess the impact of anesthetic drugs on intraoperative evoked potential, detected no randomized controlled trials (RCTs) [23]. There are only a few studies on the effects of desflurane on FVEPs under current monitoring conditions and the effect of different inhalation concentrations of desflurane on FVEPs.

Therefore, we aim to conduct a randomized controlled trial to compare the effects of low inhaled concentration of desflurane combined with propofol-remifentanil-balanced anesthesia versus high inhaled concentration of desflurane combined with remifentanil anesthesia maintenance on the amplitude and latency of intraoperative FVEPs monitoring. We hypothesize that a low inhaled concentration of desflurane combined with propofol-remifentanil-balanced anesthesia would be more suitable for monitoring FVEPs during spinal surgery.

## Methods

**Fig. 1:**
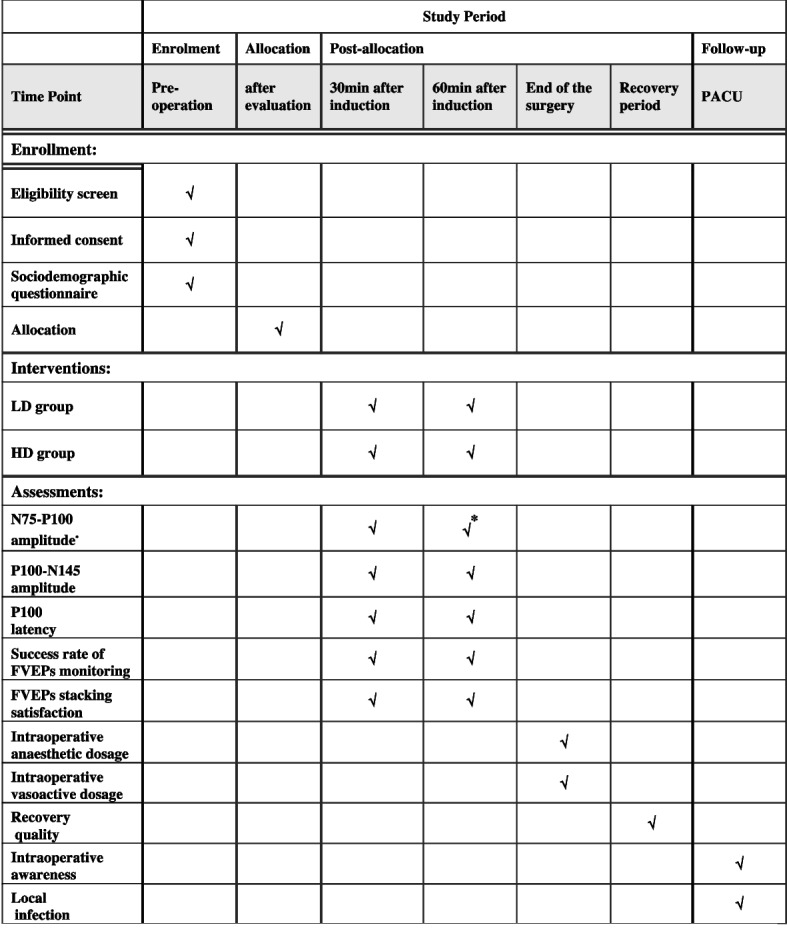
Schedule of enrollment, intervention, and assessment. Asterisk symbol (*) indicates primary outcome; LD, low inhaled concentration of desflurane combined with propofol-remifentanil-balanced anaesthesia; HD, high inhaled concentration of desflurane combined with remifentanil anaesthesia; FVEPs, flash visual evoked potentials; PACU, post-anaesthesia care unit

This protocol was prepared and presented following the Standard Protocol Items, and the enrolment, intervention, and assessment schedules are summarized in Fig. 1.

### Study design and setting

In this prospective, randomized, controlled, open-label, patient-assessor-blinded and superiority trial, patients will be screened and recruited consecutively at Beijing Tiantan Hospital, Capital Medical University.

### Ethics and dissemination

The Ethical Committee of Beijing Tiantan Hospital, Capital Medical University approved the study on July 11, 2022 (KY2022-056–02). The study was registered on clincaltrials.gov on July 15, 2022 (NCT05465330). The first patient was recruited for the study on July 20, 2022; the estimated study completion date is June 1, 2024. In addition, it complies with the principles of the Helsinki Declaration, and the protocol (V1.0; April 8, 2022) is written in accordance with the SPIRIT 2013 guidelines (Supplementary Material 1).

The Good Clinical Practice (GCP) office of our hospital will perform an audit every 12 months, and the process will be independent from investigators and the sponsor. The Data Monitoring team in our research group is responsible for managing the day-to-day conduct of the trial and data management. Plans for communicating significant protocol modifications (e.g., changes to eligibility criteria, outcomes, analyses) to relevant parties (e.g., investigators, trial participants, trial registries, journals, regulators) will be submitted to the Ethics Committee and WHO Trial Registration system in time for approval before any subsequent studies are carried out. The study findings are meant to be published in peer-reviewed journals and presented at national or international conferences.

### Participants

The designated anesthesiologist in our team will participate in the recruitment of patients scheduled for elective spine surgery under general anesthesia in this trial. Inclusion criteria are as follows: (1) patients aged 18–65 years old; (2) American Society of Anaesthesiologists (ASA) status I-III grade; (3) willingness to sign written informed consent. Exclusion criteria are the following: (1) preoperative visual impairment; (2) severe liver and kidney disease; (3) history of asthma, uncontrolled chronic disease, such as high blood pressure, diabetes, unstable angina, or mental illness; (4) body mass index (BMI) ≥ 30 kg/m^2^; (5) abuse of analgesics and history of drug abuse; (6) allergy to silicone or rejection of visual evoked potential monitoring before surgery.

### Randomization and blinding

**Fig. 2:**
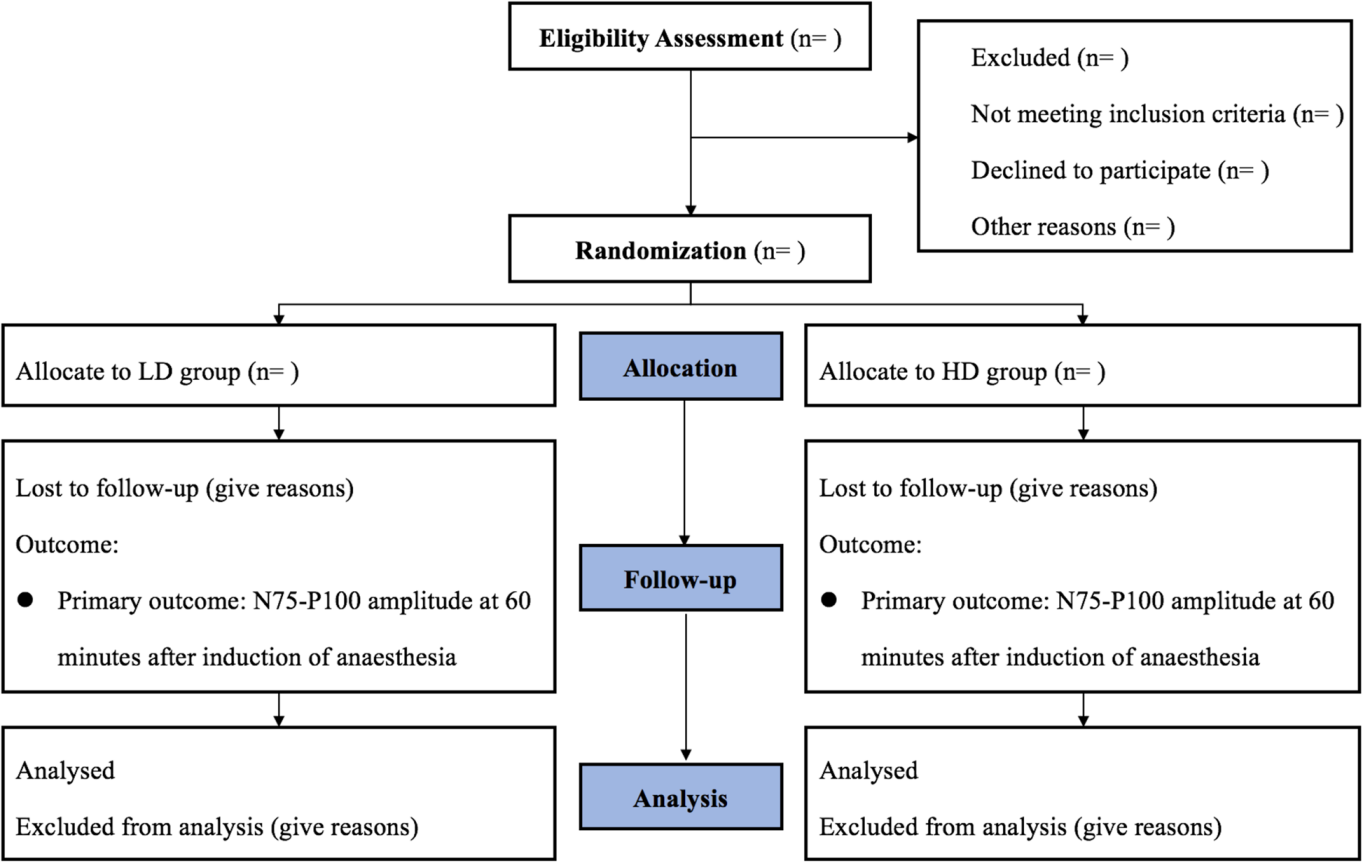
Flow diagram: the schedule of enrollment, interventions, and assessments. LD, low inhaled concentration of desflurane combined with propofol-remifentanil-balanced anesthesia; HD, high inhaled concentration of desflurane combined with remifentanil anesthesia

The patients undergoing elective spinal surgery will be randomly assigned to the LD or HD groups (Fig. 2). Eligible subjects or their legal representatives will be required to sign the written informed consent prior to randomization and surgery by the trained assessor of our team (for the flow chart, see Fig. 2). Supplemental file 1 introduces the details of the patient’s informed consent.

Randomization will occur on the date of spine surgery. Block randomization will be conducted to allocate patients to the LD group or HD group at a 1:1 ratio. The randomized digital table will be generated by an independent researcher using SPSS 25 and will be placed in a closed, opaque envelope. A designated person not involved in anesthesia management or follow-up will perform the assignment randomization sequence to ensure blinding. After the patient enters the operating room, the attending anaesthesiologist will open the sealed envelope to obtain the assignment information.

The neuroelectrophysiologists (primary outcome assessors), neurosurgeons, follow-up assessors, and biostatisticians will be blinded to the grouping; however, the anaesthesiologists will be aware of the specific assignments, as the interventions in this trial require different anesthesia protocols. A designated experienced neuroelectrophysiologist will monitor intraoperative FVEPs for all patients.

### Anaesthesia regimen

No pre-induction drugs will be administered to avoid latent interference effects with FVEPs. Baseline parameters will be monitored perioperatively, including blood pressure, electrocardiogram (ECG), pulse oxygen saturation, bispectral index (BIS), and body temperature. Anesthesia induction and maintenance of propofol will be conducted with a target-controlled infusion (TCI) device (Marsh model, Master TCI-Diprifusor, Fresenius, Brezins, France). After 5 min of preoxygenation, general anesthesia induction will be performed by sequence TCI propofol 5 μg/ml, bolus of remifentanil 1–2 μg/kg, and rocuronium 0.6 mg/kg intravenously to complete tracheal intubation. Respiratory parameters will be adjusted to a tidal volume of 8 ml/kg and a respiratory rate of 10–12 breaths/min; PaCO_2_ will be maintained at 35–45 mmHg. After induction of anesthesia, all patients will be maintained with TIVA (propofol 3–4 μg/ml plasma concentration and remifentanil 0.05–0.2 μg/kg/min) for 30 min to obtain baseline FVEP values. Next, for the patients in the HD group, we will administer desflurane (0.7–1.0 MAC of end-tidal concentration) and remifentanil (0.05–0.2 μg/kg/min) for maintenance, while desflurane (0.5 MAC) will be combined with propofol (1.5–2.5 μg/mL) and remifentanil (0.05–0.2 μg/kg/min) in the LD group. The plasma concentration of propofol will be adjusted in the LD group, and the MAC of desflurane will be titrated between 0.7 and 1.0 to maintain the BIS (BIS Vista monitor, Aspect Medical Systems, Natick, MA) between 40 and 50, primarily focusing and avoiding burst suppression. The mean arterial pressure (MAP) and heart rate (HR) will be maintained at ± 10% compared with baseline. The anesthesiologist will give vasoactive drugs such as urapidil, dopamine, esmolol, and atropine if the blood pressure and heart rate fluctuate >  ± 10%. Intraoperative body temperature will be maintained between 36 and 37°C.

### Intervention

An ophthalmologist will examine all patients to ensure normal visual function one day before the surgery. The patients will also undergo intraoperative neurophysiological monitoring using electrodes placed subcutaneously or in a corkscrew, including FVEPs and electroretinography (ERG) monitoring (to ascertain efficient delivery of retinal light stimulation).

**Fig. 3:**
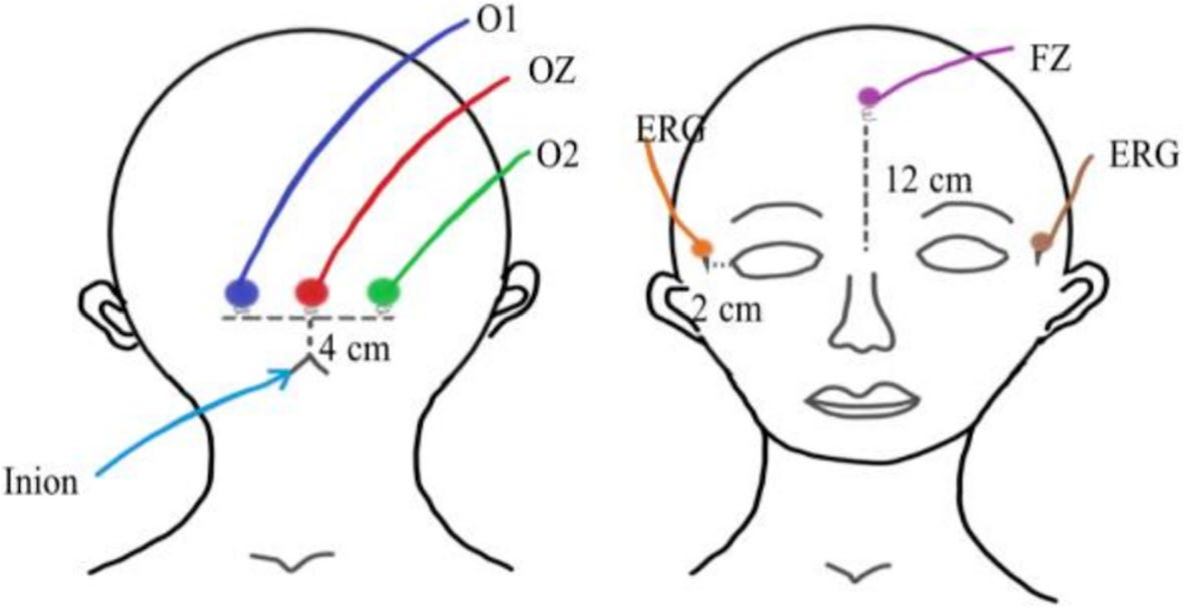
Schematic diagram of electrode position for flash visual evoked potential monitoring. OZ, 4 cm above the occipital trochanter; O1, 4 cm left lateral to the OZ; O2, 4 cm right lateral to the OZ; FZ, midfrontal area, 10–12 cm above the nose. ERG, electroretinography

The corkscrew electrodes for FVEPs will be located in the occipital zero (OZ) (4 cm above the occipital trochanter), O1 (4 cm left lateral to the OZ), O2 (4 cm right lateral to the OZ), frontal zero (FZ) (midfrontal area, 10–12 cm above the nose), and 2 ground electrodes. Electrodes for ERG will be placed 2 cm from the lateral canthus of the patient’s eyes (Fig. 3). FVEP stimulation, data recording, and storage will be performed using the Medtronic NimEclipse neuromonitoring system, which provides goggles with 6 embedded light-emitting diode (LED) bulbs. The specific parameters will be as follows: white light source high-intensity LED, 1.1 Hz stimulation frequency, 5000 to 10,000 lx laser emission, smoothing bandpass 20 to 100 Hz, and 1 to 100 Hz filter bandpass [21–23]. Each pulse stimulation will last 10 ms. The same experienced neuroelectrophysiologist will monitor intraoperative FVEPs for all procedures. We named the vertical distance between N75 and P100 the N75-P100 amplitude and the vertical distance between P100 and N145 the P100-N145 amplitude and defined P100 as the latency of FVEPs in our study. We will measure the baseline amplitude and latency of FVEPs 30 min after anesthesia induction under TIVA in both groups, which will be marked as N75-P100 (TIVA), P100-N145 (TIVA), and P100 (TIVA). Then, anesthesia maintenance with the HD group or LD group will be implemented according to the randomization results. We will measure the amplitude and latency of FVEPs 60 min after anesthesia induction in LD or HD group, which will be accordingly marked as N75-P100 (LD), P100-N145 (LD), and P100 (LD) vs N75-P100 (HD), P100-N145 (HD), and P100 (HD). At each chosen time point, we will measure the N75-P100 amplitude, P100-N145 amplitude, and P100 latency 3 times and record the average of the measurements.

### Remedy

If the FVEPs fail to record, neurophysiologists will check the stimulating apparatus and conditions, such as the stimulus intensity of light, intervals, and waves of ERG, to ensure light conduction and ascertain efficient delivery of retinal light stimulation [17]. The anaesthesiologists will check physiological parameters such as blood pressure, body temperature, and positioning. If an ideal FVEP waveform cannot be obtained after excluding all the above reasons, the surgeon will decide whether to continue the operation. Finally, if the FVEPs fail to record or the surgery is suspended, the patients will remain in the study, but the event will be recorded on the case report form.

### Study endpoints

FVEP stimulation, data recording, and storage will all be performed using the Medtronic NimEclipse neuromonitoring system. The same experienced neuroelectrophysiologist will monitor intraoperative FVEPs for all procedures.

The primary endpoint of this study is the N75-P100 amplitude at 60 min after the induction of anesthesia, which is defined as the mean N75-P100 amplitude on O1, O2, and Oz leads in both eyes.

The secondary endpoints are as follows:

1. N75-P100 amplitude decline rate compared to baseline measured under TIVA, calculated by the formula $\frac{\left[N75-P100\left(TIVA\right)-N75-P100\left(HD\right)\right]}{\left[N75-P100\left(TIVA\right)\right]}\times100\%$ or $\frac{\left[N75-P100\left(TIVA\right)-N75-P100\left(LD\right)\right]}{\left[N75-P100\left(TIVA\right)\right]}\times100\%$. N75-P100 (TIVA) will be measured 30 min after anesthesia induction under TIVA in both groups. N75-P100 (HD) or N75-P100 (LD) will be measured 60 min after anesthesia induction in HD or LD group.
2. P100-N145 amplitude decline rate compared to baseline measured under TIVA, calculated by the formula $\frac{\left[P100-N145\left(TIVA\right)-P100-N145\left(HD\right)\right]}{\left[P100-N145\left(TIVA\right)\right]}\times100\%$ or $\frac{\left[P100-N145\left(TIVA\right)-P100-N145\left(LD\right)\right]}{\left[P100-N145\left(TIVA\right)\right]}\times100\%$. P100-N145 (TIVA) will be measured 30 min after anesthesia induction under TIVA in both groups. P100-N145 (HD) or P100-N145 (LD) will be measured 60 min after anesthesia induction in HD or LD group.
3. Rate of prolongation of P100 latency compared to TIVA baseline measurements, calculated by the formula $\frac{\left[P100\left(HD\right)-P100\left(TIVA\right)\right]}{\left[P100\left(TIVA\right)\right]}\times100\%$ or $\frac{\left[P100\left(LD\right)-P100\left(TIVA\right)\right]}{\left[P100\left(TIVA\right)\right]}\times100\%$. P100 (TIVA) will be measured 30 min after anesthesia induction under TIVA in both groups. P100 (HD) or P100 (LD) will be measured 60 min after anesthesia induction in HD or LD group.
4. The success rate of FVEP monitoring is defined as the ratio of the number of eyes with a satisfactory waveform to the number of all monitored eyes.
5. FVEP stacking satisfaction will be assessed by electrophysiologists. A satisfactory waveform that could be evoked no more than three times will be considered satisfactory.
6. Intraoperative anesthetic and vasoactive drug dosage, including anesthetics (i.e., sufentanil, remifentanil, propofol, rocuronium) and vasoactive agents (i.e., atropine, ephedrine, noradrenaline, phenylephrine, urapidil)
7. Recovery quality:
  Respiratory recovery time is defined as the time from the cessation of anesthesia to the patient’s spontaneous breathing.
  Eye-opening time is the time from anesthesia cessation to when the patient can be called to open their eyes.
  Extubation time is defined as the time from the cessation of anesthesia to the removal of the patient’s tracheal catheter.
  Post-extubation agitation score. Ratings will be performed immediately after awakening and 15, 30, and 60 min after awakening: 1 point—restful sleep; 2 points—awake and calm; 3 points—irritability or irritability or crying; 4 points—difficulty to comfort or uncontrollable crying; 5 points—unable to be quiet or confused or delirium.
  Post-extubation sedation score. Ramsay sedation scale will be administered to the patients immediately after awakening and at 15, 30, and 60 min after awakening: 1 point—awake but anxious and restless; 2 points—cooperation, orientation, quiet; 3 points—drowsiness and response to commands; 4 points—drowsiness, rapid response to tapping eyebrow or loud auditory stimuli; 5 points—lethargy, slow response to tapping eyebrow or loud auditory stimuli; 6 points—drowsiness, no response at all. Scores 2–4 indicate satisfactory sedation and 5 to 6 excessive sedation.

### Follow-up

Potential perioperative adverse events of this research protocol will be followed up and evaluated in the PACU 2 h after surgery by an anaesthesiologist blinded to the assignment, which will include intraoperative awareness and local infection in electrode monitoring. Definitions and relevant plans are as follows:

1. Intraoperative awareness: regaining consciousness during general anesthesia. Close monitoring of the BIS during surgery to prevent BIS values from exceeding 60. If such a situation occurs, the anesthesia will be immediately deepened.
2. Local infection: strict aseptic procedures should be followed.

### Reporting of adverse events

All adverse events associated with this trial will be collected systematically and closely monitored until resolution or stabilization or until it has been shown that study treatment is not the cause of the event. The principal investigator of our team is responsible for reporting all adverse events to the Ethical Committee system of Beijing Tiantan Hospital. If adverse events occur, they will be immediately reported to the research department, and the principal investigator will be informed to determine the severity of the adverse events and decide whether to reveal the participant’s allocated intervention or terminate the trial according to the severity. All detected adverse events will be reported in trial publications. The definition of specific adverse events is listed at the above follow-up section.

### Data management

Dedicated persons will manage all the data. We intend to use the dual measure evaluation, dual data entry, and statistical analysis by double trained assessors to ensure the reliability and accuracy of the results. They will photograph all paper versions of the original materials and store them in an encrypted database. All electronic data will be stored in the electronic medical system of Beijing Tiantan Hospital. The electronic data of FVEPs will be saved in the Medtronic NimEclipse neuromonitoring system by the same neuroelectrophysiologist.

Patients’ personal information will be appropriately hidden, and only research-related data will be retained for statistical analysis. Paper materials, including the study protocol, case report form, and informed consent form, will be placed in a dedicated locked cabinet.

### Sample size calculation

We used PASS 15 to calculate the sample size for this study. According to the pre-experiment results, at 60 min after induction of anesthesia, the amplitudes of the LD group and HD group were 3.43 ± 0.78 μV and 2.37 ± 0.80 μV, respectively. According to the results of other studies, the superiority margin was defined as 20% of the N75-P100 amplitude in the HD group [6]. According to *α* = 0.025 and *β* = 0.2, a sample size of 63 cases can provide a superiority margin of 20% higher amplitude in the LD group than in the HD group. We conservatively set the dropout rate at 10% on the basis of actual 1.7% in our previous study, the sample size should be a total of 70 persons will be enrolled, and 35 patients in each group.

### Statistical analysis

All data analyses will be performed on both a per-protocol (PP) and intention-to-treat (ITT) basis. The intent-to-treat population will include all subjects randomized to receive HD or LD anesthesia maintenance, and the completed FVEP data will be collected. The per-protocol population will include all subjects in the ITT population not identified as protocol violators (i.e., change in the anesthesia strategy after the trial initiates). These protocol violations will be shown in a listing. Subjects who do not obtain available FVEP data or abandon the study for various reasons will be considered to have failed HD/LD and will be excluded from any form of analysis. For the missing data, the last observation and the worst-case imputation scenarios will be used as the main interpolation method.

The statistical analysis will be performed by an independent statistician using SPSS V.25.0 (Somers, NY, USA). Histograms and the Shapiro‒Wilk test will be used to assess the normality of the data distribution. For the statistical description, the continuous variables will be presented as the mean (standard deviation, SD) for normally distributed data or median (interquartile range, IQR) for skewed distributions. Categorical variables will be reported as counts and percentages, and the relative risk with its 95% confidence interval (CI) will be calculated. The absolute standard deviation (ASD) will be determined to identify any imbalance in baseline characteristics.

The primary outcome (N75-P100 amplitude of FVEPs recorded 1 h after induction) will be analyzed using a superiority test. According to the results of other studies, the superiority margin was defined as 20% of the N75-P100 amplitude in the HD group [6].For secondary outcomes, *χ*^2^ tests or Fisher’s exact tests will be performed for categorical variables including N75-P100 amplitude decline rate, P100-N145 amplitude decline rate, rate of prolongation of P100 latency, success rate of FVEP monitoring, FVEP stacking satisfaction, adverse events related to FVEP monitoring, and recovery quality. Anaesthetic and vasoactive drug agent consumption will be analyzed using independent samples *t*-tests for normally distributed variables and Wilcoxon sum tests for variables with skewed distributions. The study will terminate after all the data for the last patient are collected.

A 1-sided *P* value of 0.025 will be considered statistically significant for the primary outcome. If the confidence interval for mean differences of N75-P100 amplitude between LD and HD groups lied above the lower limit of *δ* with *P* < 0.025, we defined LD group will be superior to HD group. While all *P* values will be 2-sided with a significant level of 0.05 for the comparisons of N75-P100 amplitude decline rate, P100-N145 amplitude decline rate, rate of prolongation of P100 latency, success rate of FVEP monitoring, FVEP stacking satisfaction, anaesthetic and vasoactive drug agent consumption, and adverse events related to FVEP monitoring or recovery quality between the group LD and HD.

## Discussion

This randomized, controlled, patient- and assessor-blinded, and superiority clinical trial aims to assess the superiority effect of low inhaled concentration of desflurane combined with propofolremifentanil-balanced anesthesia versus high inhaled concentration of desflurane combined with remifentanil anesthesia on intraoperative FVEP monitoring. During flash light stimulation, the latencies of FVEPs in healthy eyes and eyes with visual impairment are widely distributed and overlap considerably [24, 25]. Therefore, using amplitude as an assessment and predictor is more meaningful than latency change. Thus, the primary outcome in this study is the N75-P100 amplitude of the FVEPs 1 h after the induction of anesthesia.

Neuromonitoring is usually performed under TIVA, which is also true for FVEPs. However, TIVA may not be suitable for lengthy surgeries needing motor-evoked potential monitoring with strict restriction for muscle relaxant supplements, especially during long-term spine surgery or neurosurgery. Intravenous-inhalation combined anesthesia has the advantage of synergistically acting on GABA and NMDA receptors that reduce the dosage of anesthetics and weaken the suppression of FVEPs. However, its impact on intraoperative FVEP monitoring has not been conclusive, and to the best of our knowledge, there are only a few relevant studies. In 2017, the Uribe study first proposed that compared with TIVA, desflurane-propofol balanced anesthesia greatly reduces the amplitude of FVEPs and prolongs their latency [16]. However, this study has many limitations. First, a combination of drugs was used for the balanced anesthesia group, including dexmedetomidine, remifentanil, and propofol. The doses of each drug were not clear. The synergistic effect of the combination of multiple drugs may aggravate the effects on FVEPs. Second, the range of BIS values was not strictly limited. Third, the small sample size (*n* = 19) may produce false positive or negative results. In 2021, we conducted a noninferiority study, which showed that the effect of sevoflurane 0.5 MAC combined with propofol and remifentanil-balanced anesthesia on the P100-N145 amplitude was not inferior to that of the TIVA group [17]. In our previous study, two groups of patients were controlled within the same BIS range combined with ERG monitoring, and the success rate of FVEP monitoring was 100%. Thus, our conclusion suggests a new anesthesia approach of sevoflurane-propofol balanced anesthesia maintenance for FVEPs besides TIVA. Yet, the effect of inhalation at different concentrations on FVEPs remains unclear. Desflurane has the advantage of high anesthesia recovery quality [22]. We hypothesize that desflurane-propofol balanced anesthesia can benefit the monitoring of FVEPs during spinal surgery compared with desflurane alone.

Wiedemayer et al*.* observed unstable waveforms of FVEPs in which the P100 latency was prolonged by 8–16%, and the P100-N145 amplitude was reduced by 60–67% under TIVA [26, 27]. In the present study, patients in the control group will receive desflurane-remifentanil anesthesia instead of total intravenous anesthesia. Due to the unique pharmacological advantages of desflurane with a low blood-gas partition coefficient and high quality of anesthesia recovery, a desflurane-remifentanil anesthesia maintenance strategy has been increasingly used in neurosurgery anesthesia [21]. In addition, there is no clinical data on the effect of desflurane inhalation anesthesia on intraoperative FVEPs. Also, an appropriate anesthesia regimen for FVEPs to protect visual function during surgery is an ongoing topic of interest among researchers.

The present study has a rigorous neuromonitoring and anesthesia regimen. In addition to anesthetic drugs, intraoperative monitoring of FVEPs is affected by hypothermia, hypotension, deep anesthesia, monitoring equipment, preoperative vision, surgical position, and surgical operations, such as drilling and flipping skin flaps [28]. This trial will be subject to rigorous inclusion and exclusion criteria, and a standardized process will be set up. In order to avoid visual function bias, all patients will be examined by an ophthalmologist to ensure normal visual function 1 day before the surgery. We will adjust the plasma concentration of propofol in the LD group and titrate the MAC of desflurane between 0.7 and 1.0 to strictly maintain the BIS between 40 and 50, primarily focusing and avoiding burst suppression. FVEPs will be monitored during quiet moments without any surgical manipulation. An intraoperative thermal blanket will be used to maintain axillary temperature between 36 and 37°C. Moreover, if intraoperative blood pressure exceeds the range of ± 10% of basal blood pressure, the anaesthesiologist will use vasoactive drugs, such as dopamine and urapidil, to maintain hemodynamic stability. FVEP stimulation, data recording, and storage will be performed using the Medtronic NimEclipse neuromonitoring system by the same experienced neuroelectrophysiologist.

In summary, this randomized, controlled, superiority trial aims to evaluate the suitability of low concentration of desflurane combined with propofol-remifentanil-balanced anesthesia for monitoring FVEPs during spinal surgery. The expected result is that low inhaled concentration of desflurane combined with propofol-remifentanil-balanced anesthesia is superior to high inhaled concentration of desflurane combined with remifentanil anesthesia for intraoperative FVEP monitoring. This study is expected to provide high-quality evidence for the effect of desflurane on FVEPs, aiming to explore a new feasible anesthesia scheme for the clinical application of FVEPs and visual function protection.

## Trial status

The protocol version number and date: The study was registered on clincaltrials.gov on July 15, 2022 (NCT05465330).

Approval for the study was certified by the Ethical Committee of Beijing Tiantan Hospital, Capital Medical University, on July 11, 2022 (KY-2022–056-02).

The date recruitment began:

The study recruited the first patient on July 20, 2022.

The approximate date when recruitment will be completed:

The estimated study completion date is June 1, 2024.

### Supplementary Information

Supplementary Material 1: SPIRIT 2013 Checklist.

Supplementary Material 2: Human Subjects Research Checklist.

## Data Availability

The full protocol, participant datasets, and statistical code used and/or analyzed during the current study are available from the corresponding author on reasonable request.

## Abbreviations

FVEPs: Flash visual evoked potentials
LD: Low inhaled concentration of desflurane
HD: High inhaled concentration of desflurane
TIVA: Total intravenous anesthesia
MAC: Minimum alveolar concentration
SSEP: Somatosensory evoked potential
MEP: Motor-evoked potential
RCTs: Randomized controlled trials
GABA: Gamma-aminobutyric acid receptors
NMDA: N-methyl-D-aspartic acid receptors
PACU: Post-anesthesia care unit
GCP: Good Clinical Practice
ASA: American Society of Anaesthesiologists
BMI: Body mass index
ECG: Electrocardiogram
BIS: Bispectral index
TCI: Target controlled infusion
MAP: Mean arterial pressure
HR: Heart rate
ERG: Electroretinography
OZ: Occipital zero
FZ: Frontal zero
LED: Light-emitting diode
ITT: Intent-to-treat
PP: Per-protocol
SD: Standard deviation
IQR: Interquartile range
95% CI: 95% Confidence interval
ASD: Absolute standard deviation
